# Umbilical Cord-Derived Mesenchymal Stem Cells Are Able to Use bFGF Treatment and Represent a Superb Tool for Immunosuppressive Clinical Applications

**DOI:** 10.3390/ijms21155366

**Published:** 2020-07-28

**Authors:** Lenka Tesarova, Klara Jaresova, Pavel Simara, Irena Koutna

**Affiliations:** 1 International Clinical Research Center, St. Anne’s University Hospital, Pekarska 53, 65691 Brno, Czech Republic; k.schichelova@gmail.com (K.J.); pavel.simara@fnusa.cz (P.S.); qkoutna@fnusa.cz (I.K.); 2 Department of Histology and Embryology, Faculty of Medicine, Masaryk University, Kamenice 5, 62500 Brno, Czech Republic

**Keywords:** mesenchymal stem cells, umbilical cord, advanced therapy medicinal product, immunosuppression

## Abstract

Mesenchymal stem cells (MSCs) have become a promising tool in cellular therapy for restoring immune system haemostasis; however, the success of clinical trials has been impaired by the lack of standardized manufacturing processes. This study aims to determine the suitability of source tissues and culture media for the production of MSC-based advanced therapy medicinal products (ATMPs) and to define parameters to extend the set of release criteria. MSCs were isolated from umbilical cord (UC), bone marrow and lipoaspirate and expanded in three different culture media. MSC phenotype, proliferation capacity and immunosuppressive parameters were evaluated in normal MSCs compared to primed MSCs treated with cytokines mimicking an inflammatory environment. Compared to bone marrow and lipoaspirate, UC-derived MSCs (UC-MSCs) showed the highest proliferative capacity, which was further enhanced by media supplemented with bFGF, while the cells maintained their immunosuppressive characteristics. Moreover, UC-MSCs expanded in the bFGF-enriched medium were the least sensitive to undesirable priming-induced changes in the MSC phenotype. Surface markers and secreted factors were identified to reflect the cell response to inflammatory priming and to be variable among MSCs from different source tissues. This study demonstrates that UC is a favorable cell source for manufacturing MSC-based ATMPs for immunosuppressive applications. UC-MSCs are able to use the bFGF-enriched medium for higher cell yields without the impairment of immunosuppressive parameters and undesirable phenotype changes after inflammatory preconditioning of MSCs before transplantation. Additionally, immunosuppressive parameters were identified to help finding predictors of clinically efficient MSCs in the following clinical trials.

## 1. Introduction

MSCs are adult stromal cells of mesodermal origin with the ability of self-renewal, multipotent differentiation and secretion of paracrine factors [1,2]. In addition, MSCs are capable of modulating immune system responses, whereby the cells themselves are low immunogenic. Because of these unique properties, MSC have become a promising tool for advanced cellular therapies in regenerative and immunomodulation applications.

There is no unique marker for MSC identification, and the general definition summarizes several shared characteristics [1,3]. MSC express CD105, CD73 and CD90, lack the expression of hematopoietic markers (CD45, CD34, CD14 or CD11b, CD79a or CD19 and HLA class II), form colonies and differentiate into osteoblast, adipocyte and chondroblast in vitro. These minimal MSC criteria were defined by Mesenchymal and Tissue Stem Cell Committee of the International Society for Cellular Therapy (ISCT) [3]. Cells bearing MSC characteristics were first derived in 1968 from bone marrow (BM) [4], which is still regarded as the gold standard MSC source. Since then, MSCs have been isolated from multiple organs and tissues of the human body including adipose tissue (AT), UC, dental pulp, placenta, etc. [5,6,7,8]. Although fulfilling ISCT minimal criteria, MSCs have been reported to exhibit heterogeneity between different source tissues and donors in surface markers expression, proliferation capacity, trilineage differentiation potential, cell qualities after differentiation and immunomodulation abilities [9,10,11,12]. The most common MSC sources with the clinical potential are BM, UC and AT. BM-derived MSCs (BM-MSCs) are still the most used, although their disadvantages include: highly invasive donation procedure, low cell proliferation capacity and the decline in cell number, maximal life span and differentiation potential with increasing donor age [13,14,15,16]. On the other hand, UC-derived MSCs (UC-MSCs) and AT-derived MSCs (AT-MSCs) are obtainable by less invasive methods and larger cell quantities are assured by high proliferation capacity of UC-MSCs and high frequency MSC content in AT [16,17]. Nevertheless, UC-MSCs and AT-MSCs have been less investigated in clinical trials compared to BM-MSCs. MSCs do not express costimulatory molecules, they are not immunogenic and do not stimulate alloreactivity [18,19,20]. This unique immunologic and survival property is one of the most important factors regarding the MSC clinical use in cell therapies, when there is no need for immunosuppressive medication after MSC transplantation [21]. Moreover, MSC-based ATMPs can be used in both autologous and allogeneic regime and there is no limitation to the source tissue available in the patient.

Regenerative applications of MSC-based ATMPs are most often focused on the regeneration of musculoskeletal, respiratory tract, central nervous system, vascular, myocardial and pancreas tissue (https://clinicaltrials.gov). In many of these conditions, differentiating and paracrine mechanisms of the MSC therapy are accompanied by the suppression of inflammation, when the immunomodulatory effects of MSC are manifested. In an inflammatory environment, MSCs induce surface adhesion molecules by which they contact immune cells and facilitate paracrine immunomodulation through secreted factors and exosomes. Thanks to that, there is a multiple effect on immune system including the inhibition of T-, B- and NK-cell proliferation, the prevention of B-cell terminal differentiation, the reduction of antigen presenting cell functions or the activation of Treg cells [19,22,23,24,25]. These MSC abilities are used to restore immune system haemostasis in immunosuppressive clinical applications. To improve the clinical potential of MSCs, priming approaches have been introduced to empower the cells. In immunosuppressive applications the treatment with pro-inflammatory cytokines and growth factors are used. Interferon gamma (IFN-y) or the combination of IFN-y and tumor necrosis factor alpha (TNF-α) are the most often used according to the ISCT recommendations [26] to increase the immunosuppressive effects of MSCs [27,28,29,30]. Extended combinatory strategies of other cytokines include interleukin-1 beta, interleukin-6 (IL-6), interleukin-17, interleukin-23, basic fibroblast growth factor (bFGF) or transforming growth factor beta (TGFβ). Besides immunocyte suppression, these combinations have been found to increase cell migration to inflammatory sites, secretion of trophic factors, adhesion to extracellular matrix and vascularization in vivo [31,32,33]. Other priming approaches, such as priming with other molecules [34,35,36,37], hypoxia [38,39,40], biomaterials and specific culture conditions [41,42,43] are also applicable in regenerative applications. After these kinds of priming, MSCs have been characterized by increased survival, migration, secretion, angiogenic, and differentiation properties thanks to which they induced better regeneration and function of tissues in vivo. 

According to the ClinicalTrials.gov database there are 228 clinical trials conducted around the world (active, recruiting, or enrolling by invitation status) subjecting MSC-based ATMPs (June 2020, https://clinicaltrials.gov). Twenty-seven of them are related to immune system disorders including graft versus host disease and autoimmune diseases, such as Crohn’s disease, rheumatoid arthritis, multiple sclerosis, asthma, etc. The safety and efficiency of BM-MSCs have mostly been evaluated; nevertheless UC-MSCs and AT-MSCs are investigated in some trials targeting graft versus host disease and rheumatoid arthritis. So far, only a few ATMPs have received marketing authorisation in the European Union. MSC-based ATMP, Alofisel, is one of them approved in 2018 for treatment complex perianal fistulas in adults with Crohn’s disease (https://www.ema.europa.eu/en/medicines/human/EPAR/alofisel). Other MSC-based ATMPs targeting immune system disorders received marketing authorization in Japan, Korea, Canada and New Zealand [44]. It is obvious that despite the number of clinical trials using MSCs that have been and are in progress worldwide, only a few of them have yielded satisfactory results. The above-mentioned source tissue and donor heterogeneity in the MSC content, proliferation capacity, differentiation potential and in vivo efficiency impairs clinical upscaling of the MSC-based ATMPs production. The solution is to use different source tissues and priming approaches for different clinical applications. However, this is conditioned by sorting out well all steps of the production process, including (i) source tissue selection, (ii) MSC derivation, (iii) MSC priming, (iv) MSC expansion, (v) product characterization and formulation, and (vi) product application in different condition types. From the clinically relevant source tissues it is possible to harvest only a limited number of MSCs that require ex vivo expansion to reach the demanding therapeutic dosage. Large-scale MSC expansion was facilitated by bioreactor devices, including stirred tank [45,46,47], rocking [48], fixed-bed [49] and hollow fiber [50,51] bioreactors. The cell culture takes place automatically in a close system, which makes the production more reliable and consistent and less contamination risky when the number of manipulations and open events is significantly reduced. Production of MSC-based ATMPs is not very demanding in terms of culture media. Both MSC derivation and expansion is performed in simple media based on Alpha modified Eagle’s minimum essential medium or Dulbecco’s modified Eagle’s medium (DMEM) and fetal bovine serum (FBS) or fetal calf serum [48,49,50,51]. A humanised alternative is the so-called D5 media using human platelet lysate (hPL) [52,53,54,55], while the transition to xeno-free chemically defined media is gradual for the time being [46,47]. There is no need for media supplement with growth factors, nevertheless bFGF is sometimes used in both FBS-based and serum-free expansion media. In any case, it is necessary to develop the most defined and the least heterogenic expansion systems because it was suggested that ex vivo MSC expansion may affect their phenotype and therapeutic abilities [56,57,58]. Another significant weak point in the production of MSC-based ATMPs are release criteria. Reflecting ISCT minimal criteria, they are limited to some of the following: sterility, viability, karyotype, colony formation, trilineage differentiation and basic immunophenotype identifying the cells. However, these parameters do no cover MSC heterogeneity and do not represent a potency assay which would take the clinical response in patients into account. It is necessary to identify, follow up and correlate such potency parameters with the results of clinical studies to determine predictors of MSC-based ATMP efficiency in particular clinical applications.

This study is focused on the clinical use of MSCs and addresses the shortcomings in the production of MSC-based ATMPs that impair the results of recent clinical trials. MSCs are derived from clinically relevant source tissues, UC, BM and lipoaspirate. Production of MSC-based ATMPs is simulated in three different culture media, established for MSC expansion. The resulting cells are characterized in normal state and after priming with pro-inflammatory cytokines to mimic an inflammatory environment. MSC proliferation capacity, phenotype and immunosuppressive abilities are evaluated with the aim to formulate recommendations on how to best use the established MSC-based ATMP production system, to determine the suitability of source tissues and culture media and to define parameters to be specified in immunomodulatory applications of MSCs and correlated with their clinical efficiency. Moreover, our study is expected to bring the UC closer to the cGMP production of MSC-based ATMPs by confirmation that this source tissue has the potential to produce larger batches that may be in stock for repeated administration and to reach immunosuppressive parameters comparable to more commonly used BM-MSCs. 

## 2. Results

### 2.1. Derivation and Characterization of Tissue-Specific MSCs

**Figure 1:**
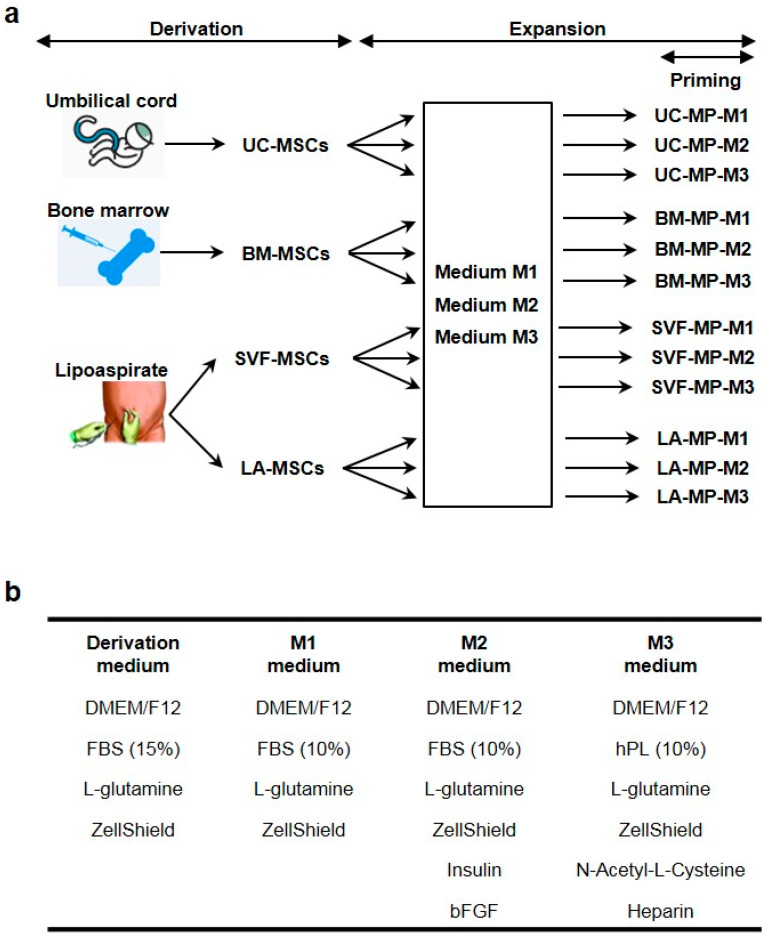
Experiment scheme. (**a**) MSCs were derived from UC, BM and lipoaspirate fractions and expanded in three different media. After expansion tissue-specific MPs were characterized in normal and primed state. (**b**) Derivation and expansion media content, component concentrations are given in the Materials and Methods section.

**Figure 2:**
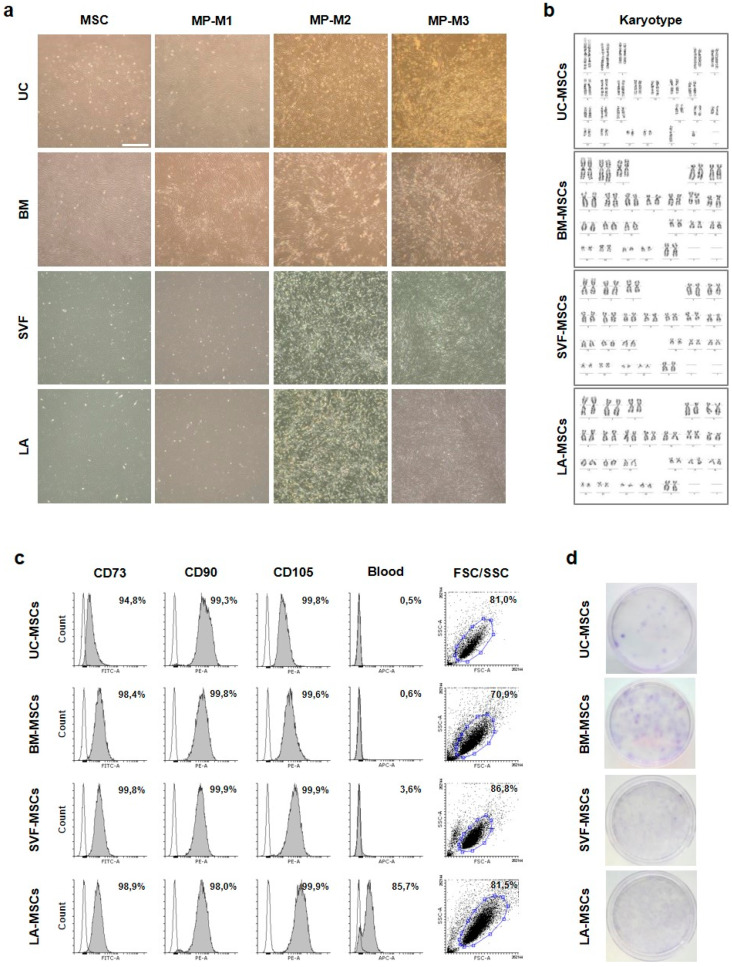
Characterization of tissue-specific MSCs. (**a**) Morphology (phase-contrast image) of adherent cells during MSC derivation and MPs after three passages in M1, M2 and M3 media. Scale bar, 1 mm. (**b**) Derived MSCs have normal karyotype. Chromosome numbers were counted in at least 40 spreads and at least 10 of them were fully differentiated at 450–500 G-band level. (**c**) Flow cytometry results for MSC markers. MSCs were positive for CD73, CD90 and CD105 and negative for blood markers. The grey area represents the marker expression, the grey line depicts isotype control expression, the percentage of marker positive cells is given. FSC/SSC dot plot denotes the gated population. (**d**) CFU-F ability of MSCs seeded at the density of 50 cells per square centimeter. Giemsa staining shows variable number and potency of MSC progenitors.

The cells were derived from UC, BM and two fractions of lipoaspirate, blood and adipose fraction (Figure 1a, Materials and Methods section). During derivation adherent cells were spindle shaped with clear nucleus growing in monolayer. Cells from BM and lipoaspirate fractions were widely dispersed while cells from UC were smaller and grew densely side by side (Figure 2a). At 80–90% confluence tissue-specific MSCs were harvested, UC-MSCs were derived from UC, BM-MSCs from BM, SVF-MSCs from blood fraction (stromal vascular fraction) of lipoaspirate and LA-MSCs from adipose fraction of lipoaspirate (Figure 1a).

Derived MSCs displayed normal karyotype (Figure 2b) and were characterized by expression of MSC marker combination of CD73, CD90 and CD105 higher than 95% (Figure 2c). The ratio of cells positive for blood markers CD14, CD19, CD34 and CD45 did not exceed 4% with the only exception of LA-MSCs which expressed CD34. Clonogenicity of MSCs was confirmed by the formation of colony-forming unit–fibroblastic (CFU-F) with differences between tissues (Figure 2d). Many potent progenitors manifested by dark colonies were detected in BM-MSCs. Many progenitors were found also among SVF- and LA-MSCs, but lighter colonies suggested their weaker potential. UC-MSCs were characterized by the lowest number of progenitors forming darker colonies.

Multipotency of tissue-specific MSCs was confirmed by trilineage differentiation, nevertheless differences were found between source tissues in adipogenic and osteogenic potential (Figure S1). UC-MSC-derived adipocytes were characterized by many lipid droplets of a uniform small size, while BM-MSCs differentiated into sporadic adipocytes with big lipid droplets. Many adipocytes were differentiated from SVF- and LA-MSCs with large amounts of lipid droplets showing a considerable size distribution. The greatest osteogenic potential was detected in BM-MSCs. The activity of alkaline phosphatase was detected even in some undifferentiated MSCs and rapidly increased during differentiation. BM-MSC-derived osteoblasts were characterized by huge amounts of calcium deposits similar to LA-MSCs, while UC-MSCs were less productive. Chondrogenic differentiation was comparable between samples confirmed by aggrecan and collagen II staining. 

Taken together, MSCs from four different tissues and tissue fractions were derived and characterized by expression of MSC markers. Tissue-specific differences were detected in the ability to form CFU-F and the trilineage differentiation potential.

### 2.2. The Highest Proliferative Capacity of UC-MSCs Is Further Enhanced in M2 Media

**Figure 3:**
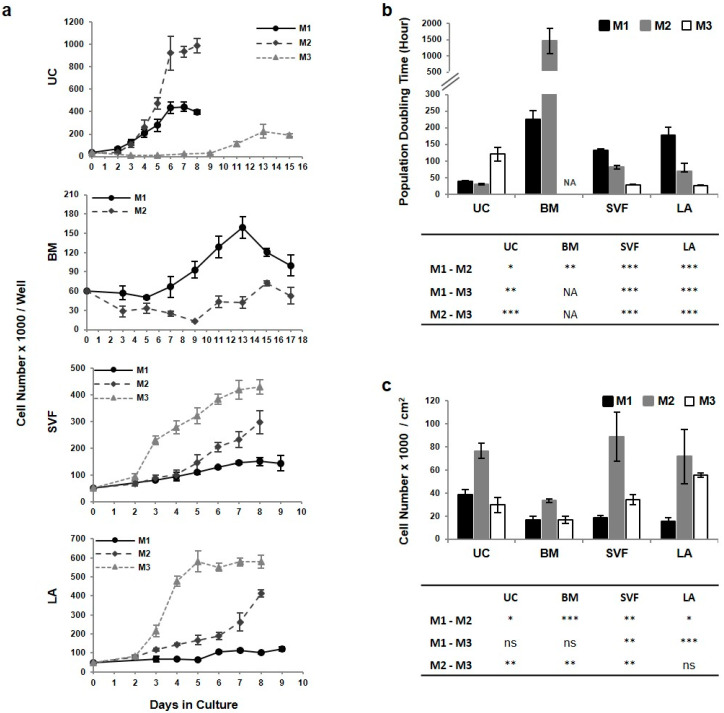
Growth characteristics of tissue-specific MSCs. During the third passage of tissue-specific MSCs in M1, M2 and M3 media, the growth curve was established (**a**) and population doubling time was calculated (**b**). (**c**) The yield of MSCs harvested at the 80–90% confluency. Results refer to the mean ± SD, *n* = 3. Tables in (**b**,**c**) show the significance level of difference between expansion media within MSC lines. Statistics: unpaired t test (* *p* < 0.05, ** *p* < 0.01, *** *p* < 0.001; ns, not significant). NA—not available.

Tissue-specific MSCs were expanded in three different media for three passages, which simulated the manufacturing of medicinal product (MP). The cells at the point of the MP harvest and analyzing the specifications were labelled with “MP” along with the abbreviations for source tissue and expansion media (e.g., UC-MP-M1, Figure 1a). The cells in other experiment phases (derivation, expansion and post MP cultivation until replicative senescence) were simply referred to as MSCs. During the third passage, the growth curve was established and population doubling time (PDT) was calculated (Figure 3b). Variability in the cell growth dynamics was observed among MSC lines from different tissues and media. Some lines were characterized by a typical sigmoid pattern of proliferation, while the growth curve of others displayed gradual increase without clearly defined logarithmic growth phase. In UC-MSC the shortest PDT was detected in M2 media, in SVF- and LA-MSCs the shortest doubling time was induced by M3 media and exhaustion of the proliferative capacity was detected in BM-MSCs. In M1 media, PDT was the longest among all MSC lines, in M2 the cells proliferate extremely slowly with PDT, almost 1500 h, and it was not possible to determine PDT at all in M3 media.

During the MSC expansion cell morphology changes were observed among expansion media (Figure 2a). In M1 media there were spindle shaped widely dispersed cells, while in M2 media the cells were much smaller and not so dispersed along the culture surface. In M3 media differences were detected among MSC lines. While UC- and BM-MSCs were extended with thin cell body and long processes, SVF- and LA-MSC were small with rounded cell body and short thin processes. The morphology variability was also reflected in the cell yield expressed by the number of cells per square centimeter at 80–90% confluence (Figure 3c). In all MSC lines M2 media was the most yieldable.

**Figure 4:**
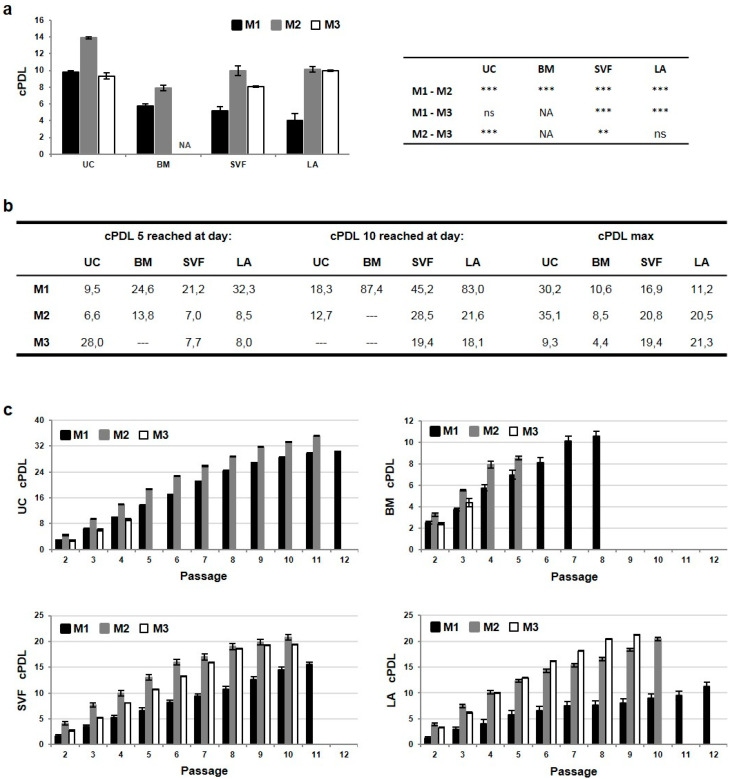
cPDL of tissue-specific MSCs. (**a**) cPDL of MSC lines expanded in M1, M2 and M3 media after three passages at the time of MSC-based MP harvest. (**b**) Proliferation rate of MSC lines expressed as the day when cPDL level of 5 and 10 was achieved. Maximal cPDL (cPDL max) reached by MSC lines at replicative senescence. (**c**) The cPDL increase during the culture period of MSC lines. Results refer to the mean ± SD, *n* = 3. Table in (**a**) shows the significance level of difference between expansion media within MSC lines. Statistics: unpaired t test (** *p* < 0.01, *** *p* < 0.001; ns, not significant). NA—not available.

During expansion cumulative population doubling level (cPDL) of MSC lines was monitored. At the time of MP harvest, cPDL ranged between 4 and 14 depending on the MSC line and expansion media (Figure 4a). Thanks to the shortest PDT and the highest cell yield, the highest cPDL was detected in UC-MP cells in M2 media. The proliferation rate of MSC lines was expressed as the day when cPDL level of 5, respective 10, was achieved (Figure 4b). The fastest proliferation was induced by M2 media in UC-MSCs followed by SVF-, LA- and BM-MSCs. cPDL level during long-term culturing was analyzed until replicative senescence to assess the length of culture period (Figure 4c). The shortest culture period was observed in BM-MSCs especially in M3 media, where the culture was exhausted as early as after the second passage and no BM-MP-M3 could have been harvested and characterized. In M2 and M1 media BM-MSCs were able to replicate to the fifth and eighth passage, respectively, and reached cPDL between 8 and 10. On the contrary, UC-MSCs in M1 and M2 media replicate up to eleventh or twelfth passage and reached cPDL over 30. Similar profile was detected for SVF- and LA-MSCs. In M1 media the cells replicate until the twelfth passage, in M2 media the cells were exhausted earlier but reached much higher cPDL levels over 20. Compared to UC- and BM-MSCs, there was significantly different culture period in M3 media.

Taken together, UC-MSCs were characterized by the highest proliferative capacity, which was further enhanced by addition of bFGF and insulin to the media. M2 media induced shorter PDT and higher number of cells per square centimeter, so the higher cell yield was achieved in a shorter time, compared to M1. Moreover, UC-MSCs reached the highest maximal cPDL and related overall cell yield. Significant variability in the length of culture period was observed among MSC lines expanded in M3 media. 

### 2.3. Priming of MSCs Expanded in M2 Media Interferes with Cell Proliferation, CFU-F Ability and CD90 Expression

**Figure 5:**
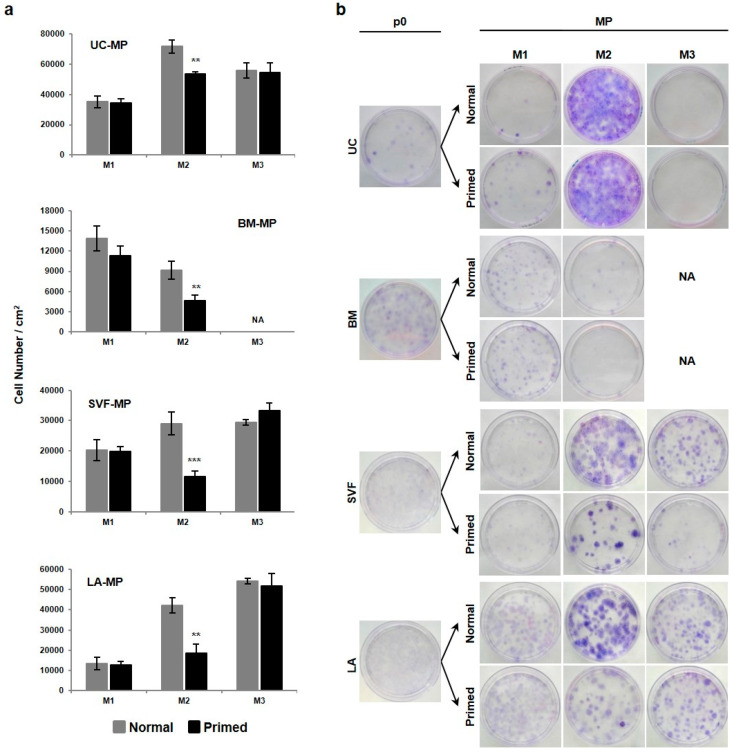
Proliferative properties of tissue-specific MSC-based MPs. (**a**) The yield of normal and primed cells expanded in M1, M2 and M3 media at the time of MP harvest. (**b**) CFU-F ability of normal and primed MPs seeded at the density of 50 cells per square centimeter. Giemsa staining shows variable progenitor number and potency. Results refer to the mean ± SD, *n* = 3. Statistics: unpaired *t* test (** *p* < 0.01, *** *p* < 0.001). NA—not available.

After three passages in M1-M3 media tissue-specific MPs were harvested and their proliferative and immunosuppressive properties were evaluated. This evaluation was performed on normal cells as well as on cells after pro-inflammatory priming with IFN-γ a TNF-α (Figure 1a). This MSC priming was used to mimic inflammatory site in vitro and to analyze cell behavior in such a simulated environment. According to our preliminary experiments (data not shown) priming was applied to cells at approximately 50% confluence for 72 h. After this time, the number of cells was determined (Figure 5a) and it was found that the effect of priming was uniform across MPs from all tissues. In M1 and M3 media the cell number was the same in normal and primed MPs, while in M2 media it decreased by priming. MSC surface markers were analyzed in MPs to assess the effect of expansion and priming on the stability of MSC lines (Table 1). Stable CD73 and CD105 expression over 90% was detected in both normal and primed MPs. In contrast, expression of CD90 dropped to 53–82% after priming in MSC lines expanded in M2 media and also to 85% in normal BM-MP-M2.

The clonogenicity of MPs was determined by CFU-F analysis (Figure 5b). CFU-F ability of normal MPs reflected the above-mentioned proliferative capacity of MSCs lines. Many potent UC-MSC progenitors were preserved during expansion in M2 media compared to a few in M1 and none in M3. The number of BM-MSC progenitors detected after derivation decreased during expansion in M2, while survived in M1. In contrast, SVF- and LA-MSC progenitors were preserved during the expansion in all three media and moreover became more potent in M2 and M3. CFU-F analysis of primed MPs revealed the decrease of MSC progenitor numbers in BM-MP-M2, SVF-MP-M2, LA-MP-M2 and SVF-MP-M3 after priming.

To test the possibility to reverse the effect of M2 media on the MSC phenotype after priming, the M2-M1 switch was performed (see Material and Methods). Decreased cell numbers observed after priming in M2 media were suppressed by the M2-M1 switch; nevertheless, the cell yield of M2-M1 switched MSCs dropped to the level of MSC in M1 media (Figure S2b). Similarly, during the CFU-F analysis, the number of UC- and LA-MSC progenitors did not decrease after priming thanks to the M2-M1 switch, but the potency of surviving progenitors was lower compared to M2 medium (Figure S2c). Expression of CD90, which dropped in M2 media to 14–53% in primed MSC and to 84–89% in normal BM- and LA-MSC, was normalized to the values above 98% in both normal and primed MSC after M2-M1 switch (Table 1). At the same time, M2-M1 switched MSCs were characterized by stable CD73 and CD105 expression over 95%. Regarding the cell yield, maximal cPDL level decreased in M2-M1 switched MSCs (more significantly in BM- and LA-MSC) compared to MSCs left in M2 media; however, the decreased cPDL was still much higher compared to MSCs cultivated continuously in M1 media (Figure S2a).

To sum up, MPs expanded in M2 media are more sensitive to priming, which is manifested by decreased cell proliferation, lower ratio of MSC progenitors and weaker expression of CD90 marker. Our data showed that UC-MPs are the most resistant to the negative effects imposed by priming. The switch of MSC expanded in M2 medium into M1 medium for one passage was sufficient to rescue MSC phenotype. However, this was at the expense of lower cell expansion during the switched passage.

### 2.4. Priming of MSC Induces Expression of Immunosuppressive Surface Molecules

**Figure 6:**
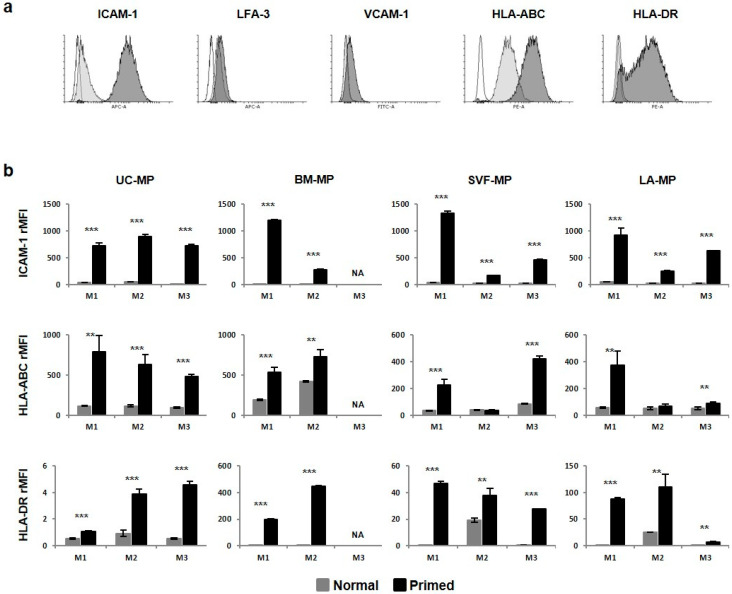
Flow cytometry analysis of adhesion and HLA molecules in tissue-specific MSC-based MPs. (**a**) Representative histograms show expression of ICAM-1, LFA-3, VCAM-1, HLA-ABC and HLA-DR in normal (colored light grey) and primed (colored dark grey) MPs versus isotype control (black line). (**b**) Results are expressed as relative mean fluorescence intensity (rMFI) and compared between normal and primed MPs expanded in M1, M2 and M3 media. Results refer to the mean ± SD, *n* = 3. Statistics: unpaired *t* test (** *p* < 0.01, *** *p* < 0.001). NA—not available.

Normal and primed tissue-specific MPs were subjected to flow cytometry analysis of adhesion and HLA molecules. Results were analyzed for the ratio of positive cells and relative mean fluorescence intensity (rMFI) for each molecule and both parameters were found to increase after priming (Figure 6a). The ratio of ICAM-1^+^ increased significantly across all samples over 96% with the exception of UC-MP-M3 and BM-MP-M2 where it was 76% and 86% respectively (Table 1). Weaker induction was detected for LFA3 and VCAM-1 markers, whereas the highest positivity was detected in cells from M1 medium. The ratio of HLA-ABC^+^ cells was over 95% in all normal and primed samples with the exception of BM-MP-M2 with 70% positivity. No HLA-DR^+^ cells were detected in normal MPs, except approximately 50% positivity in both lipoaspirate-derived MPs in M2 media. The percentage of HLA-DR cells increased in all samples after priming. This increase was stronger (over 90%) in BM-MP-M1, SVF-MP-M1 and M2 and LA-MP-M1 and -M2, in other samples the percentage did not exceed 20%. rMFI was used to compare molecule expression intensity between samples. The most significant priming-induced rMFI increase and variability was detected for ICAM-1, HLA-ABC and HLA-DR (Figure 6b). Primed UC-MPs were characterized by similar ICAM-1 rMFI levels in all three media, while in primed MPs from other tissues the highest rMFI level was in M1. Expression of HLA-ABC in primed samples was much intense in UC- and BM-MPs compared to both lipoaspirate MPs. In the case of HLA-DR, there were significant rMFI differences among MPs from different tissues. UC-MPs were characterized by the lowest rMFI, followed by SVF-, LA- and BM-MPs with the highest rMFI.

Taken together, MSC priming induced adhesion and HLA molecules in terms of both the ratio of positive cells and rMFI. The most significant priming-induced expression increase was detected for ICAM-1 and HLA molecules. There were irregular differences among MPs from different tissues; nevertheless, UC-MPs were characterized by smaller variability between expansion media while in other tissues MPs from M1 media exceeded MPs from M2 media in expression of several molecules. 

### 2.5. MSCs Respond to Priming by Increased IDO Activity and IL-6 Production

Immunosuppressive paracrine activity of tissue-specific MPs was determined before and after priming including following parameters: indoleamine 2,3-dioxygenase (IDO) activity and secretion of bFGF, hepatocyte growth factor (HGF), IL-6 and TGFβ1. IDO activity was determined through the amounts of its metabolite, kynurenine, in culture supernatant. A standard curve of kynurenine determined the detection limit to 0.5–75 µg/mL and allowed to quantify the amount of kynurenine in MP samples. No IDO activity was detected in normal MPs, but it was induced by priming. All primed samples depleted tryptophan and converted it into kynurenine which concentration in culture supernatant ranged from 10.0 to 13.5 µg/mL. 

**Figure 7:**
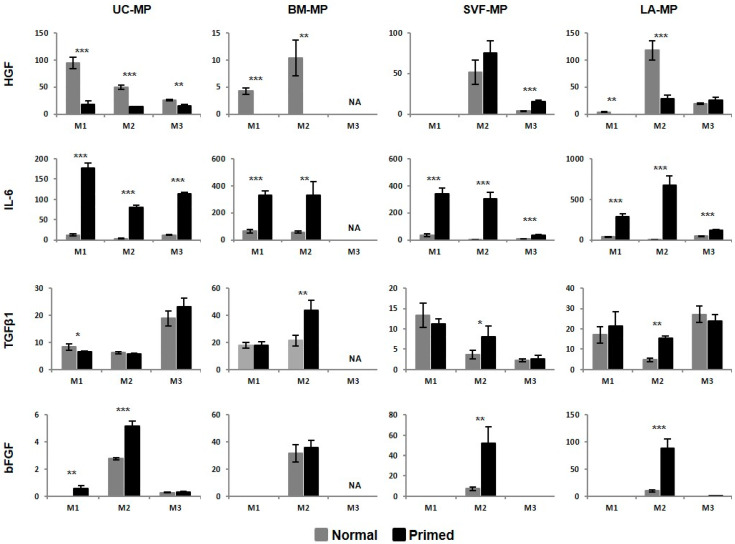
Paracrine activity of tissue-specific MSC-based MPs. Cytokine secretion in normal and primed MPs given in nanograms per million cells. Results refer to the mean ± SD, *n* = 3. Statistics: unpaired *t* test (* *p* < 0.05, ** *p* < 0.01, *** *p* < 0.001). NA—not available.

Compared to IDO activity, cytokine secretion was detected in both normal and primed MPs (Figure 7). Normal UC-MP-M1 were characterized by high HGF production (almost 100 ng/million cells) compared to MPs from other tissues (only up to 5 ng/million cells) where increased HGF secretion was detected in M2. After priming, HGF production dropped to the levels below 30 ng/million cells. Production of IL-6 was low in normal MPs, up to 64 ng/million cells and increased after priming, up to 114 ng/million cells in UC-MPs, 332 ng/million cells in BM-MPs, 340 ng/million cells in SVJ-MPs, and 671 ng/million cells in LA-MPs. The smallest differences between normal and primed MPs were detected for TGFβ1. Only in BM-MP-M2, SVJ-MP-M2, and LA-MP-M2 production of TGFβ1 increased after priming. There were no differences among MP-M1 from different tissues with TGFβ1 level between 15 and 20 ng/million cells. bFGF2 production was detected in normal MPs expanded in M2 and it increased after priming. The highest production was characteristic for BM-MPs before priming and LA-MPs after priming. Additionally, low FGF2 secretion (up to 0.9 ng/million cells) was detected in normal UC-MP-M3 and primed UC-MP-M1, UC-MP-M3 and LA-MP-M3. Concentration of the above-mentioned cytokines was determined also in fresh expansion media. The only detectable cytokine amounts were as follows: 14.9 ng of FGF2/mL in M2, 1.1 ng of TGFβ1/mL in M1, 1.5 ng of TGFβ1/mL in M2, and 5.6 ng of TGFβ1/mL in M3. 

To sum up, MSC secretome analysis documented reaction of tissue-specific MPs to priming. Primed MPs were characterized by increased IDO activity and IL-6 production and decreased HGF production. Addition of FGF2 to culture media induced secretion of this factor by cultivated cells and this secretion increased after priming.

### 2.6. MSCs from all Source Tissues Are Able to Suppress T-Cell Proliferation

**Figure 8:**
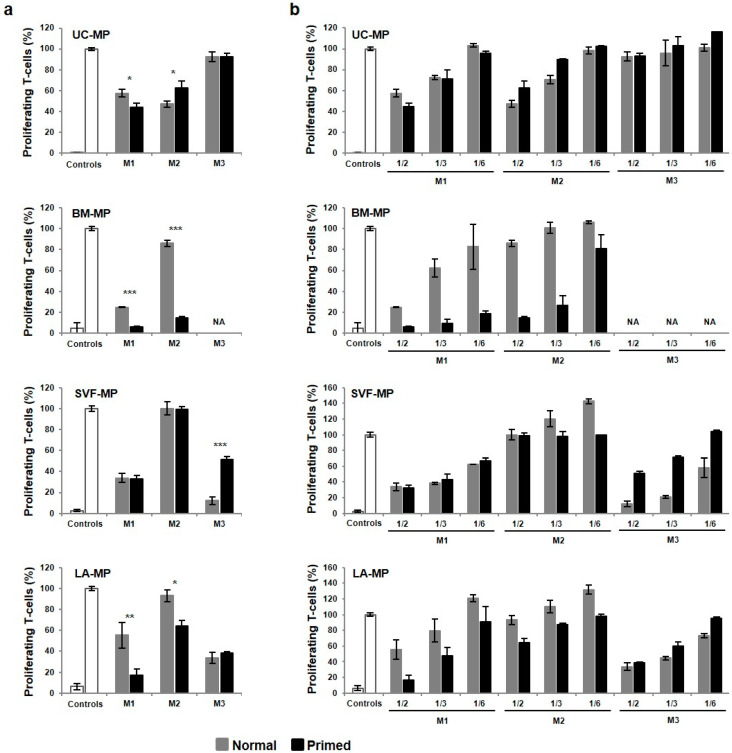
Immunosuppressive ability of tissue-specific MSC-based MPs. The ratio of CFSE-labelled proliferating T-cells after co-cultivation with MPs normalized to stimulated T-cells (right white control column); unstimulated T-cells, left white control column. (**a**) Proliferation of T-cells suppressed by normal and primed MPs expanded in M1, M2 and M3 media. (**b**) Dose-dependency of the suppressive effect evaluated by seeding MPs in three different concentrations: cell number of PBMC/MP = 1/2, 1/3 and 1/6. Results refer to the mean ± SD, *n* = 3. Statistics: unpaired t test (* *p* < 0.05, ** *p* < 0.01, *** *p* < 0.001). NA—not available.

Immunosuppressive ability of MPs was evaluated by in vitro mixed lymphocyte reaction (MLR). First, normal MPs were analyzed (Figure 8a, grey columns). Among cells expanded in M1 media BM-MPs were the most suppressive, in M2 media UC-MPs were the best and in M3 media only MPs from both lipoaspirate fractions worked well. In terms of expansion media, UC-MPs from M1 and M2 media were comparable, while BM-, SVF- and LA-MPs from M1 were more suppressive compared to M2. Second, primed MPs were assessed (Figure 8a, black columns). Immunosuppressive effect was strengthened by priming in only some samples, most significantly in BM-MP-M1 and M2, when the ratio of proliferating T-cells dropped to 6 and 14%, respectively. For MLR MPs were seeded in three different concentrations to evaluate dose-dependency of the suppressive effect (Figure 8b). Both normal and primed MPs from all tissues and media were characterized by a dose-dependent gradual suppressive effect with the only exception of primed SVF-MP-M2. It is important to point out the fact that instead of suppression, some samples of low concentrated MPs supported proliferation of T-cells. The ratio of proliferating T-cells over 110% was found especially in normal MPs from lipoaspirate fractions in M2 media.

Taken together, the best immunosuppressive effect was detected in primed BM-MPs while UC-MP-M3, SVJ-MP-M2 and LA-MP-M2 were almost unsuppressive. In M2 media, only UC-MPs retained M1 medium-like immunosuppressive properties, while MPs from other tissues suppressed better in M1 media. Effective MPs were characterized by dose-dependent suppression of T-cell proliferation, which suggests cell contact-dependent mechanism of action.

## 3. Discussion

The immunomodulatory and regenerative potential of MSCs, together with their low immunogenicity, have been known for decades and evaluated in hundreds of clinical trials worldwide. Despite this, clinical upscaling of MSC-based ATMP production still suffers from the heterogeneity of the source tissues and the cells themselves, unintegrated production process and basic release criteria for MSC characterization. In our study, we addressed this problem by covering source tissue variability and evaluated the effect of culture media composition on MSC phenotype, proliferation fitness and cell yield and defined parameters to be correlated with in vivo MSC potency in immunomodulatory clinical applications.

MSCs were derived from clinically relevant source tissues: BM, the traditional MSC source, UC and lipoaspirate. It is necessary to point out that frozen bone marrow was used for the MSC derivation. Nevertheless, it has been reported in the literature that cryopreservation does not affect BM-MSC morphology, expression of surface markers, or proliferation and differentiation potential [59]. However, the effects on viability, migration, attachment, genomic stability and paracrine secretion remain undefined due to the variability in the cryopreservation process and to the lack of standardized assays. After derivation, the production of MSC-based ATMP was simulated by cell expansion in media M1, M2 and M3. These media were based on FBS (M1 and M2), which is the standard growth supplement during manufacturing, and on hPL (M3), the most common FBS alternative [48,49,50,51,52,53,54,55]. M2 medium was further supplemented by insulin and bFGF. UC-MSCs were characterized by the highest proliferative capacity with the shortest PDT and long culture period. Before the cells became senescent, they reached cPDL over 30. MSC proliferation rate was further enhanced by addition of bFGF and insulin to the media. The higher cell yield was achieved in a shorter time, when M2 media induced shorter PDT and a higher number of cells per square centimeter. Lower proliferative capacity was detected in MSCs from lipoaspirate and BM-MSCs were characterized by the lowest proliferation potential that was quickly exhausted. Significant variability in the length of culture period was observed in tissue-specific MSC lines expanded in M3 media. The sensitivity of cells to the hPL heterogeneity is one of the arguments why to replace FBS by chemically defined serum-free media when refining protocols for MSC-based ATMP manufacturing [46,47]. The recommended MSC dose range in immunosuppressive applications is 0.5–2.5  ×  10^6^ cells/kg body weight per intravenous infusion and it is similar for local injections [60,61]. If starting with 500,000 MSCs after the derivation phase, clinically relevant cPDL varies from 6.5–9 per infusion. For UC-MSCs this is only a third of the maximal cPDL and it is reached within 2–3 passages in M1 medium and 1.5–2 passages in M2 medium. For MSCs from lipoaspirate it is the half of the maximal cPDL and for BM-MSCs clinically relevant cPDL nearly reaches the maximal cPDL itself. Regarding the tissue-specific MSC yield, we determined that UC-derived MSCs are characterized by the fastest cell expansion, the best proliferation fitness at the moment of clinically relevant cPDL and the highest cell yield in the course of the overall culture period. These advantageous characteristics are further enhanced by the medium enrichment with bFGF and insulin. This should be considered, when it is necessary to achieve a prescribed cell dose in a specified time frame or a limited number of passages to preserve cell genome integrity. UC-MSCs have previously been characterized by short cell-doubling times, high proliferative rates and great proliferative limits [62,63]. bFGF supplementation of the UC-MSC culture has been described to promote the population growth [64] and along with the low seeding density has been found optimal for the clinical-scale expansion of UC-MSCs [65]. Direct comparison of UC-MSC growth properties with BM- and AT-MSCs under the same expansion conditions confirmed the UC-MSC superiority [16,66], with no differences between source tissues being detected [67].

There is no unique surface marker for MSCs and the characterization is still based on the multiple marker expression [3]. In our study, we determined positivity for CD73, CD90 and CD105 and negativity for blood markers CD14, CD19, CD34, CD45 and HLA-DR. These criteria were fulfilled by MSCs derived from all source tissues with the only exception of the CD34 positivity in LA-MSCs. Temporary LA-MSC expression of CD34 has been previously described [68] and in our study it was lost during the first passages and was no longer detectable in LA-MPs. Basic MSC immunophenotype was maintained during the cell expansion into MPs reflecting the cell line fitness. Only in a proliferatively exhausted BM-M2-MSC line the expression of CD90 decreased below 85% and this loss of MSC identity was also accompanied by decreased expression of HLA-ABC. MSC characterization traditionally includes trilineage differentiation into osteocytes, adipocytes and chondrocytes. Nevertheless, with the increasing diversity of MSC sources, it has been found that the differentiation potential in vitro is variable among MSCs from different source tissues. Differentiation to osteoblasts is most evident in BM-MSCs, while adipocyte formation is most extensive in AT-MSCs and low in UC-MSCs and chondrogenic differentiation is characterized by the smallest differences between source tissues or slight predominance of BM-MSCs [9,16,67,69,70]. This variability was also confirmed in our study especially for adipogenic and osteogenic differentiation. BM-MSCs were characterized by fast and effective generation of osteoblasts while MSCs from lipoaspirate exceeded in adipocyte generation. Unlike regenerative applications, the differentiation potential does not correlate with the clinical efficacy of MSCs in immunosuppressive applications. Trilineage differentiation is not often included among the release criteria for MSC-based ATMPs and the identity of the cells is limited to MSC immunophenotype.

Tissue-specific MPs were treated by IFN-γ and TNF-α to induce pro-inflammatory priming. Priming was introduced to empower the cells during ATMP manufacturing, to increase the immunosuppressive effects of MSCs [71,72]. It also makes it possible to analyze MSC behavior and characteristics in vitro in a simulated inflammatory environment. In our study, primed MPs were compared to their normal counterpart and it was found that cells expanded in M2 media, unlike M1 and M3 media, are sensitive to priming. This sensitivity applied to MPs from all source tissues and was demonstrated by decreased cell numbers, lower CFU-F ability and weaker expression of an adhesive molecule CD90. There are no data regarding the requirement of a CD90 marker and the adhesion ability for the clinical potency of MSCs. However according to our data, if MSC-based ATMPs are produced in the presence of bFGF, we suggest withdrawing the growth factor within the last expansion passage before pro-inflammatory priming. Although it decreases the cell yield, we demonstrated that this M2-M1 switch can secure the MSC phenotype after priming. Nevertheless, among source tissues, UC-MPs demonstrated the greatest resistance to priming-induced MSC phenotype changes. Thus, with this tissue, the benefits of the bFGF-enriched medium could be used throughout the whole ATMP manufacturing process without the need for the M2-M1 switch. It has already been described that bFGF in expansion media does not compromise the nature of UC-MSCs with respect to the stem cell characteristics, differentiation potential, surface antigens and immunosuppressive capacity [64,65]. The advantage of UC-MSCs being the most resistant to bFGF-conditioned sensitivity to pro-inflammatory priming is described in our study for the first time.

As previously described [73,74], flow cytometry analysis of adhesion and HLA molecules, that are necessary for immunosuppressive effect of MSCs, revealed that the expression of ICAM-1 (CD54), LFA-3 (CD58), VCAM-1 (CD106), HLA-ABC and HLA-DR was modulated by MSC priming. Both the cell positivity and rMFI increased, the most significantly of ICAM-1 and HLA-molecules. Strong induction of ICAM-1 resulted in a fully positive cell population suggesting the potential of our samples to maintain immunosuppressive function [73,75,76]. HLA molecules were characterized by the greatest variability among MPs from different tissues. HLA-ABC was constitutively expressed in both normal and primed samples, but an rMFI increase after priming was larger in UC- and BM-MPs compared to MPs from lipoaspirate. Lower HLA-ABC expression was previously correlated with weaker immunogenicity [77], suggesting MSCs from lipoaspirate less immunogenic after priming. HLA-DR expression after priming was variable in terms of both cell positivity and rMFI. In UC-MPs the least induction was detected, while MPs from BM and lipoaspirate reached over 80 percent positivity. BM-MPs also exceeded in rMFI levels being in concordance with previous studies [73,78]. Expansion of MSCs from both lipoaspirate fractions in M2 media induced 40–50% HLA-DR positivity, which was not detected in other samples. This is in concordance with the previous study demonstrating that the addition of bFGF in the culture medium does not affect the HLA-DR expression levels in UC-MSCs [65], but in contrast with the study reporting that the use of bFGF induces HLA-DR expression up to 80% in BM-MSCs [58].

Besides surface markers, tissue-specific MPs were demonstrated to respond to pro-inflammatory priming also with respect to secreted factors targeting cells of both adaptive and innate immunity. Regarding IDO activity all tissues and expansion media generated MPs with the ability to deplete tryptophan from the culture media. This is essential for immunosuppression in the presence of pro-inflammatory cytokines [79,80]. The secretion profile of immunoregulatory cytokines differed between MPs from various source tissues, which have been already described [81,82,83]. For example, UC-MPs were characterized by the highest HGF secretion, while IL-6 was more abundant in MP supernatants from BM and lipoaspirates. The effect of priming was uniform across samples, when HGF production decreased and IL-6 increased. The most comparable between MP samples were secretion levels of TGFβ1, which was shown to be responsible for inhibition of T-cell proliferation and responses [84,85,86]. The predisposition of M2 media for MSC expansion in immunosuppressive applications was suggested by increased TGFβ1 secretion after priming. Considering supplementation of M2 medium by bFGF, its secretion was also evaluated. bFGF production by MSCs is desirable for their regenerative applications, when it promotes both tissue regeneration and survival of transplanted MSCs [87,88]. In our study, only MSCs expanded in the presence of bFGF secreted detectable levels of this growth factor and the secretion was further increased after priming, especially in MPs from lipoaspirate. This result suggests MSCs from lipoaspirate and M2 media suitable for regenerative MSC applications and the use of pro-inflammatory priming not only for immunomodulatory purposes. 

Finally, immunomodulatory properties of MPs were evaluated by MLR. The best suppression was detected in BM- followed by SVF- and LA- and UC-MPs, which suppressed T-cell proliferation to about 50%. Immunosuppressive abilities of tissue-specific MSCs described in the literature are variable. While some studies reported the inhibitory effects of UC-MSCs more prominent compared to BM- and AT-MSCs [80,89], a similar degree of immunosuppression was described for UC- and BM-MSCs in another study [90] and the immunosuppressive predominance of BM- and AT-MSCs was reported by others [90]. In our study, the variability was detected also between expansion media and in the priming effect. Our data suggest that pro-inflammatory priming can enhance the immunosuppressive ability of only some samples and not others. This response to priming discrepancy has already been described for MSCs from BM and UC [89]. In UC-MPs immunosuppressive effect was comparable in M1 and M2 media, while in MPs from BM and lipoaspirate fractions M1 medium exceeded. This result is in concordance with other studies that reported that bFGF-supplemented UC-MSCs retain their immunosuppressive ability in a cell dose-dependent manner [64,65], but in contrast with the report describing enhanced immunosuppressive potential in bFGF-cultured BM-MSCs [58]. In vitro MLR can indicate immunosuppressive abilities of MSCs; however, according to our data, the methodology suffers from several drawbacks, such as the limited accuracy of seeding given cell numbers and incomparability of various samples due to different cell size and adhesion ability, which we detected in connection with the proliferation fitness, priming and the expansion media type. Moreover, it has been reported that in vitro inhibition potential of MSCs may not always correlate with in vivo clinical response in patients. Therefore, the final conclusions of immunomodulatory properties of MSCs from different source tissues must be made based on in vivo experiments. It is also important to point out, that in our study lower MP concentration induced slight enhancement in the T-cell proliferation rate especially in MLR with MPs from lipoaspirate fractions. These results indicate that the MSC dose in clinical settings should be cautiously determined. 

Taken together, our data highlight the benefits of UC and the bFGF-enriched expansion medium for the clinical upscaling of the MSC-based ATMPs production. UC is ontogenetically the youngest clinically relevant source tissue, from which MSCs with the best proliferation fitness can be derived in sufficient quantities for clinical application requiring repeated administration. The bFGF-enriched medium increased and accelerated the cell yield during the expansion phase. This advantage was best used just in UC-MSCs that were less sensitive to changes in the MSC phenotype induced by pro-inflammatory priming. Additionally, immunosuppressive parameters analyzed in this study were in UC-MSCs resistant to bFGF media supplementation, while in MSCs from BM and lipoaspirate these parameters were often better in medium without bFGF. Immunosuppressive potential of UC-MSCs was verified by priming-induced IDO activity, cytokine secretion and expression of adhesion and HLA-molecules. Compared to BM-MSCs, the ability of UC-MSCs to suppress T-cell proliferation was only about 50%. Nevertheless, the suppressive effect was clearly MSC dose-dependent, suggesting that better immunosuppression in vivo could be achieved by higher therapeutic doses. Higher MSC doses are more accessible with well-expandable UC-MSCs that express low HLA-DR levels, suggesting weaker immunogenicity in clinical applications and decreasing the risk of alloreactivity stimulation. Nevertheless, the final decisions about the most suitable source tissue for the manufacturing of MSC-based ATMPs for individual immunosuppressive applications must be made based on in vivo preclinical and clinical trials. According to the present study, we suggest extending the set of release criteria with the following parameters: (i) parameters significantly induced by priming, expression of ICAM-1 and secretion of IDO and IL-6, to assess MSC ability to respond to priming; (ii) parameters variable between samples, expression of HLA molecules and secretion of IL-6, to correlate with clinical response with the aim to find predictors of clinically efficient ATMPs.

## 4. Materials and Methods 

### 4.1. Umbilical Cord, Bone Marrow and Lipoaspirate Samples

Samples of UC (*n* = 5), BM (*n* = 3) and lipoaspirate (*n* = 4) were obtained for this study. The middle part of the UC (15–20 cm) came from a recent childbirth at the Department of Obstetrics and Gynecology, University Hospital Brno, Czech Republic. Frozen BM (100–120 milliliters) for disposal was obtained from the Transfusion and Tissue Department, University Hospital Brno, Czech Republic. Lipoaspirate (300–400 milliliters) came from the liposuction procedure at the Department of Plastic and Aesthetic Surgery, St. Anne’s University Hospital Brno. Samples were collected after obtaining written informed consent, including the agreement that the samples can be used for scientific or research purposes in cases where they are not clinically used for the needs of the patient (the clinical reason for their use disappears) or in case of demonstrable death of the patient. All the procedures were in concordance with the amended Declaration of Helsinki. 

### 4.2. Derivation of MSCs

Tissue samples were used to derive MSCs. UC was cut in 5–6 centimeter pieces, rinsed with 1X phosphate buffered saline (PBS, Thermo Fisher Scientific, MA USA) supplemented with 6.8 IU/mL heparin (Zentiva, Czech Republic) (PBS/heparin) and cut lengthwise around the blood vessels. Resulting pieces were treated with 4 mg/mL collagenase type I, II and IV (Thermo Fisher Scientific) and 1 mg/mL hyaluronidase (Sigma-Aldrich, MO, USA) at 37 °C for two hours. After digestion the cells were scraped off the tissue using the opposite end of the pipette tip, washed by PBS/heparin and subjected to red blood cell (RBC) lysis for 5 min in lysis buffer: 150 mM ammonium chloride (Penta, Czech Republic), 10 mM potassium bicarbonate (Penta) and 0.1 mM ethylenediaminetetraacetic acid (Thermo Fisher Scientific). Resulting cells were seeded on the tissue culture dish in the derivation medium (Figure 1b): DMEM/F12 (Thermo Fisher Scientific), 15% FBS (Sigma), 2 mM L-glutamine (Thermo Fisher Scientific) and 1×ZellShieldTM (Minerva Biolabs, Germany). Frozen BM was thawed in a 37 °C water bath, diluted 1:2 in PBS/heparin and centrifuged. After PBS/heparin washing the cells were treated by RBC lysis and seeded on the tissue culture dish in the derivation medium. The lipoaspirate was diluted 1:1 in 1×PBS supplemented with 500 U/mL penicillin and 500 μg/mL streptomycin and centrifuged at 700× *g* for 5 min to separate adipose and blood fractions. The cells from blood fraction were pelleted, treated by RBC lysis and seeded on the tissue culture dish in derivation medium. The adipose fraction was washed with 1× PBS and incubated with collagenase type I at 37 °C for 1 h while shaking. The digestion was stopped by addition of 10% FBS and the cells were pelleted. After RBC lysis the cells were filtered through 70 μm cell strainer (BD Bioscience, NJ, USA) and plated on the tissue culture dish in the derivation medium.

After plating, cell cultures were maintained at 37 °C and 5% CO_2_ in humidified atmosphere. Non-adherent cells were removed with PBS washing and medium exchange at day 3 after seeding. Subsequently the medium was changed every three days and adherent MSCs were cultivated to reach 80–90% confluence. At this confluence, tissue-specific MSCs were harvested using HyQTase (GE HealthCare, IL, USA) (Figure 1a).

### 4.3. Expansion and Priming of Tissue-Specific MSCs

During the first passage, tissue-specific MSCs were seeded into three different media, M1, M2 and M3, and expanded for three passages, which simulated the manufacturing of MSC-based MPs (Figure 1a). The seeding density was 4 × 10^3^ cells per cm^2^, medium was exchanged every three days and MSCs at 80–90% confluence were passaged by HyQTase treatment. M1 medium consisted of DMEM/F12, 10% FBS, 2 mM L-glutamine and 1×ZellShieldTM; M2 medium consisted of M1 medium supplemented with 10 μg/mL insulin (Thermo Fisher Scientific) and 15 μg/mL bFGF (Miltenyi Biotech, Germany); M3 medium consisted of DMEM/F12, 10% Crux Rufa hPL (Trinova Biochem, Germany), 2 mM L-glutamine, 1× ZellShieldTM, 10 mM N-Acetyl-L-Cysteine (Sigma-Aldrich) and 1 IU/mL heparin. During the third passage, MSC priming was performed by treating the cells at approximately 50% confluence by 10 ng/mL IFN-γ (Peprotech, NJ, USA) and 15 ng/mL TNF-α (SinoBiological, People’s Republic of China) for 72 h (Figure 1a). Only half of the cells were primed to compare their characteristics with normal MSCs without priming. When harvesting the third passage, the cells were at the point of simulated MP, which was reflected in the cell labelling along with the source tissue and the expansion medium. For instance, UC-MP-M1 stands for MSCs derived from UC after the third passage in M1 medium (Figure 1a). 

The growth curve was plotted, and the PDT was determined for each MSC line during the third passage. Cells were seeded in 6-well plates at a density of 4 × 10^4^ or 6 × 10^4^ cells per well and collected 2–17 days after seeding. Live cells were counted by Countess I Automated Cell Counter (Thermo Fisher Scientific) including trypan blue exclusion test. A graph was plotted of the cell number against time points indicating the growth phases. At an inflection point of the growth curve the proliferation rate expressed by PDT was calculated using the formula *PDT = T ln2/ln (A/A0)*, where *T* is the cultivation time in hours, *A* represents final cell number and *A0* corresponds to the initial cell number. cPDL in the course of passages was used to describe the overall expansion rate and cellular age using the equation *cPDL = X + 3.322 (logY − logI)*, where *X* is the initial population doubling level, *I* represents the initial cell number seeded onto the plate, and *Y* correspond to the final cell yield. MSC lines were also cultivated after MP harvest and the maximal cPDL was determined when the cells reached replicative senescence, defined as not-increasing cell number throughout two weeks of cultivation.

In additional experiments so-called M2-M1 switch was performed. Tissue-specific MSC lines UC-MSC, BM-MSC and LA-MSC, cryopreserved after derivation, were thawed and expanded for four passages in M1 and M2 media. When plating the fourth passage, half of the cells from M2 medium were seeded into M1 medium. During the cultivation cPDL was determined and in the last passage MSCs in all media combinations were primed and the cell yield, CFU-F ability and MSC marker expression were compared between normal and primed cells.

### 4.4. Characterization of MSCs

Tissue-specific MSCs were characterized by morphological evaluation, cytogenetic analysis, CFU-F assay, flow cytometry immunophenotyping, and trilineage differentiation. Basic MSC morphology was assessed after their derivation and expansion by bright field microscopy using Olympus CKX53 inverted microscope (Olympus, Japan). Karyotype analysis was performed by Cytogenetic Laboratory Brno (Cytogenetická Laboratoř Brno, s.r.o., Brno, Czech Republic) by Giemsa-banding and microscopic examination. At least 40 metaphase spreads per sample were analyzed using “LUCIA Cytogenetics” software (Laboratory Imaging, Prague, Czech Republic) at a resolution of 450–500 bands per haploid set. Self-renewal capacity of tissue-specific MSCs and MPs was assessed by CFU-F assay. Cells were seeded at low densities and day 14 cultures were fixed with methanol (Lach-Ner, Czech Republic), washed and stained with Giemsa stain (Sigma-Aldrich) to visualize fibroblast colonies derived from single cells.

### 4.5. Immunophenotyping by Flow Cytometry

Cell surface antigen expression was detected in tissue-specific MSCs and MPs using flow cytometry. The cells were harvested by HyQTase and incubated in the dark for 30 min at 4 °C with antibodies. The data about antigens CD14 (APC, Thermo Fisher Scientific), CD19 (APC, Thermo Fisher Scientific), CD34 (APC, Thermo Fisher Scientific), CD45 (APC, Thermo Fisher Scientific), CD54 (APC, Thermo Fisher Scientific), CD58 (APC, Thermo Fisher Scientific), CD73 (FITC, Thermo Fisher Scientific), CD90 (PE, Beckman Coulter), CD105 (PE, Thermo Fisher Scientific), CD106 (FITC, Southern Biotech), HLA-ABC (PE, Thermo Fisher Scientific) and HLA-DR (PE, Thermo Fisher Scientific) were collected in a BD FACS Canto II device (BD Biosciences, San Jose, CA) and analyzed with FACSDiva (BD Biosciences) and Flowing software (Turku Centre for Biotechnology, University of Turku, Turku, Finland). During the analysis isotype controls IgG1 kappa (FITC, Thermo Fisher Scientific), IgG1 kappa (PE, Thermo Fisher Scientific), IgG1 (PE, Beckman Coulter), IgG1 (APC, Thermo Fisher Scientific) and IgG2a (PE, Thermo Fisher Scientific) were included to distinguish negative and positive expression. Besides the ratio of positive cells, fluorescence intensity was determined for each marker. Mean fluorescence intensity was normalized to the intensity of the isotype control and resulting rMFI was used for comparison between samples.

### 4.6. Trilineage Differentiation

Tissue-specific MSC lines were cultured on 20 μg/mL fibronectin (Sigma-Aldrich, USA)-coated plates in the culture medium. When reaching approximately 90% confluence, culture media was replaced with differentiation induction media and the cells were cultured another three weeks with medium exchange twice a week. Adipogenic induction media contained high-glucose DMEM (Sigma-Aldrich), 10% FBS, 2 mM L-glutamine, 1× ZellShieldTM, 400 ng/mL dexamethazon (Sigma-Aldrich), 111 ng/mL 3-isobutyl-1-methylxanthine (Sigma-Aldrich), 35.8 μg/mL indomethacin (Sigma-Aldrich) and 10 μg/mL insulin. To visualize adipocyte lipid droplets, day 21 cultures were fixed in 4% paraformaldehyde (Sigma-Aldrich) and stained with 0.3% Oil Red O (Sigma-Aldrich). Osteogenic induction media contained high-glucose DMEM, 10% FBS, 2 mM L-glutamine, 1× ZellShieldTM, 40 ng/mL dexamethazon, 8.8 μg/mL ascorbic acid (Sigma-Aldrich) and 2.16 μg/mL β-glycerophosphate (Sigma-Aldrich). Alkaline phosphatase activity was detected at day 14 cultures fixed with 4% paraformaldehyde by Alkaline Phosphatase Blue Microwell Substrate (Sigma-Aldrich) according to the manufacturer’s instructions. The presence of calcium deposits was evaluated at day 21 cultures fixed in 4% paraformaldehyde by 2% Alizarin Red S staining. Chondrogenic differentiation was induced by StemProTM Chondrogenesis Differentiation Kit (Thermo Fisher Scientific) and its efficiency was evaluated after a 21-day cultivation period. Aggrecan presence was visualized by 0.1% Safranin staining and collagen II expression was detected by immunocytochemistry using Anti-Collagen Type II (20 µg/mL, Cedarlane, NC, USA) and Anti-rabbit IgG Alexa Fluor 488 (4 µg/mL, Cell Signaling Technology, Netherlands). 

### 4.7. Colorimetric Assay of Indoleamine 2,3-Dioxygenase Activity 

IDO is an enzyme that regulates the degradation of tryptophan to N-formylkynurenine, which is catabolized to kynurenine. IDO activity is therefore proportional to supernatant kynurenine levels measured spectrophotometrically. Culture supernatant from normal and primed tissue-specific MPs were collected during the MP harvest and kynurenine formation was evaluated. 100 μL of the supernatant was treated with 50 μL 30% trichloroacetic acid (Lach-Ner), vortexed and centrifuged for 10 min at 10,000× *g*. 100 μL of the obtained supernatant was transferred to a 96-well plate and an equal volume of Ehrlich’s solution (100 mg/mL p-dimethylbenzaldehyde (Sigma-Aldrich) in glacial acetic acid (Penta)) was added. Optical density was measured at 490 nm using Multiskan GO microplate reader (Thermo Fisher Scientific). The amount of kynurenine was determined using a standard curve of kynurenine (BioTechne, MN, USA) concentration ranging from 0.05 to 100 μg/mL.

### 4.8. Enzyme-Linked Immunosorbent Assay

To compare protein secretion among tissue-specific MPs, the production of bFGF, HGF, IL-6 and TGFβ1 were measured. The supernatant from both normal and primed MPs were collected 72 h after priming and the concentrations were measured with Human ELISA kits (Thermo Fisher Scientific) according to the manufacturer’s instructions using Multiskan GO microplate reader and respective standard curves. 

### 4.9. Mixed Lymphocyte Reaction

The immunomodulatory effect of tissue-specific MPs was evaluated by MLR when the suppression of T cell proliferation was determined. Peripheral blood of healthy donors was purchased from Transfusion and Tissue Department, University Hospital Brno, Czech Republic. Human peripheral blood mononuclear cells (PBMCs) were purified by a FicollTM Paque (GE Healthcare) density gradient, according to the manufacturer’s protocol, and incubated overnight at 37 °C in RPMI medium consisting of RPMI 1640 (Thermo Fisher Scientific), 10% FBS, 2 mM L-glutamine, 100 U/mL penicillin and 100 μg/mL streptomycin. The next day PBMC were labelled with 5 μM carboxyfluorescein succinimidyl ester (CFSE, CFSE Cell Division Tracker Kit, BioLegend, CA, USA) and resuspended in RPMI medium to be plated at a concentration of 1.5 × 10^5^ cells per well in a 96-well plate. T-cell proliferation was stimulated by monoclonal antibodies against CD2, CD3, CD28 using T Cell Activation/Expansion Kit (Miltenyi Biotec, Germany). 

Tissue-specific MPs were seeded in a 96-well plate at a concentration of 2.5, 5.0 and 7.5 × 10^4^ cells per well. After settling, MPs were treated with 26.3 μg/mL mitomycin C (NORDIC Pharma, Czech Republic) for 50 min to inactivate their proliferation. After mitomycin C washout stimulated CFSE stained PBMC were added to the plate and incubated for 72 h at 37 °C and 5% CO_2_ in humidified atmosphere. After this co-cultivation PBMC were harvested and incubated in the dark for 15 min with antibodies against CD4 (APC, Miltenyi Biotech) and CD8 (PE, Miltenyi Biotech) to measure proliferation of T-cells by flow cytometry. Non-stimulated CFSE stained PBMC control was used to set the gate for non-proliferating cells. The percentage of the initial T-cell population that had divided at least ones at the time of measurement was calculated. To allow comparison between different MPs, proliferation of stimulated T-cells was used for normalization.

### 4.10. Statistical Analysis

Data are presented as mean ± SD. Statistical difference was analyzed by unpaired *t* test. Statistical values less than 0.05 were considered significant (* *p* < 0.05) and values less than 0.01 and 0.001 are differentiated (** *p* < 0.01, *** *p* < 0.001).

## 5. Conclusions

In conclusion, the present work highlights the benefits of UC as a cell source for manufacturing MSC-based ATMPs for immunosuppressive clinical applications. Fast cell expansion and great proliferation fitness at the moment of UC-MSC harvesting enables the production of larger batches that may be prepared in stock for repeated administration. The required basic and immunosuppressive characteristics of UC-MSCs are resistant to expansion media changes, so it is possible to take advantage of the bFGF-enriched medium that facilitates the production process. We confirmed immunosuppressive potential of UC-MSCs, although the ability to suppress T cell proliferation in vitro was weaker compared to the still gold standard BM-MSCs. Nevertheless, the correlation of these findings with the clinical efficiency of tissue-specific MSCs is necessary to determine in clinical trials. For this purpose, we suggest several parameters to extend the set of release criteria for the cGMP MSC-based ATMP production.

## Figures and Tables

**Table 1:** Expression of MSC markers and adhesion and HLA molecules in tissue-specific MSC-based MPs. Expression was determined in normal (N) and primed (P) MPs expanded in M1, M2 and M3 media. Results refer to the mean, *n* = 3. For MSC markers, underlined numbers indicate expression below 90 per cent; for adhesion and HLA molecules, * indicates expression increase in primed MPs compared to normal MPs (unpaired *t* test, *p* < 0.05). NA—not available.

		MSC Marker Expression (%)						Adhesion and HLA Molecule Expression (%)									
		CD73		CD105		CD90		ICAM-1		LFA-3		VCAM-1		HLA-ABC		HLA-DR	
		N	P	N	P	N	P	N	P	N	P	N	P	N	P	N	P
**UC**	**M1**	90.0	98.2	99.9	99.6	100.0	99.0	37.9	99.7 *	58.8	72.6 *	0.2	8.5 *	99.9	99.9	0.3	9.8 *
	**M2**	99.4	99.5	97.7	93.5	99.4	81.9	45.7	99.9 *	29.2	23.5	0.3	4.5 *	99.0	99.7	0.7	21.0 *
	**M3**	98.4	97.3	94.8	91.0	96.1	98.1	5.9	76.3 *	16.9	39.5 *	0.1	1.3 *	96.6	99.0	0.0	29.4 *
**BM**	**M1**	99.4	99.5	99.0	98.6	100.0	100.0	21.4	97.8 *	46.3	78.2 *	17.2	32.6 *	97.9	98.6	0.0	82.9 *
	**M2**	97.2	95.5	98.9	97.6	84.6	70.2	10.4	85.8 *	19.6	22.9	5.8	5.5	69.9	74.2	0.7	18.9 *
	**M3**	NA	NA	NA	NA	NA	NA	NA	NA	NA	NA	NA	NA	NA	NA	NA	NA
**SVF**	**M1**	98.9	99.6	99.5	99.6	99.8	99.7	48.5	96.4 *	61.0	88.8 *	1.4	27.7 *	96.7	95.2	0.5	90.6 *
	**M2**	99.4	96.9	97.3	96.5	96.8	53.4	6.1	96.8 *	35.4	32.9	0.2	0.6 *	96.9	95.7	42.5	93.9 *
	**M3**	98.2	99.2	98.2	99.5	99.9	99.7	3.1	99.5 *	24.6	36.8	0.1	0.5 *	99.4	99.8	0.1	26.0 *
**LA**	**M1**	98.7	99.5	99.5	99.5	99.5	99.7	43.3	99.4 *	70.4	91.1 *	1.8	10.1 *	98.3	98.7	0.6	92.5 *
	**M2**	99.8	98.8	99.3	98.5	99.0	59.4	4.9	97.4 *	57.5	60.0	0.7	5.5 *	99.1	98.8	56.9	98.2 *
	**M3**	98.0	99.4	99.3	99.7	99.8	99.9	14.3	99.9 *	75.9	83.3 *	0.4	0.7	99.8	99.7	0.1	18.5 *

## Supplementary Materials

Supplementary materials can be found at https://www.mdpi.com/1422-0067/21/15/5366/s1. Figure S1: Trilineage differentiation potential of tissue-specific MSCs. Figure S2: Proliferative properties of tissue-specific MSCs during M2-M1 switch. Table S1: Expression of MSC markers in tissue-specific MSCs during the M2-M1 switch.

## Abbreviations

AT	Adipose tissue
ATMP	Advanced therapy medicinal product
bFGF	Basic fibroblast growth factor
cPDL	Cumulative population doubling level
BM	Bone marrow
CFSE	Carboxyfluorescein succinimidyl ester
CFU-F	Colony-forming unit–fibroblastic
DMEM	Dulbecco’s modified Eagle’s medium
FBS	Fetal bovine serum
hPL	Human platelet lysate
IDO	Indoleamine 2,3-dioxygenase
IFN-γ	Interferon gamma
IL-6	Intrleukin-6
ISCT	International Society for Cellular Therapy
LA	Adipose fraction of lipoaspirate
MLR	Mixed lymphocyte reaction
MP	Medicinal product
MSC	Mesenchymal stem cell
PBMC	Peripheral blood mononuclear cell
PBS	Phosphate buffered saline
PDT	Population doubling time
RBC	Red blood cell
rMFI	Relative mean fluorescence intensity
SD	Standard deviation
SVF	Stromal vascular fraction of lipoaspirate
TGF-β	Transforming growth factor beta
TNF-α	Tumor necrosis factor alpha
UC	Umbilical cord
