# Supplementary figures and images for: Umbilical Cord-Derived Mesenchymal Stem Cells Are Able to Use bFGF Treatment and Represent a Superb Tool for Immunosuppressive Clinical Applications
Source: Int J Mol Sci. 2020 Jul 28;21(15):5366. doi: 10.3390/ijms21155366 (PMC7432622; doi:10.3390/ijms21155366)

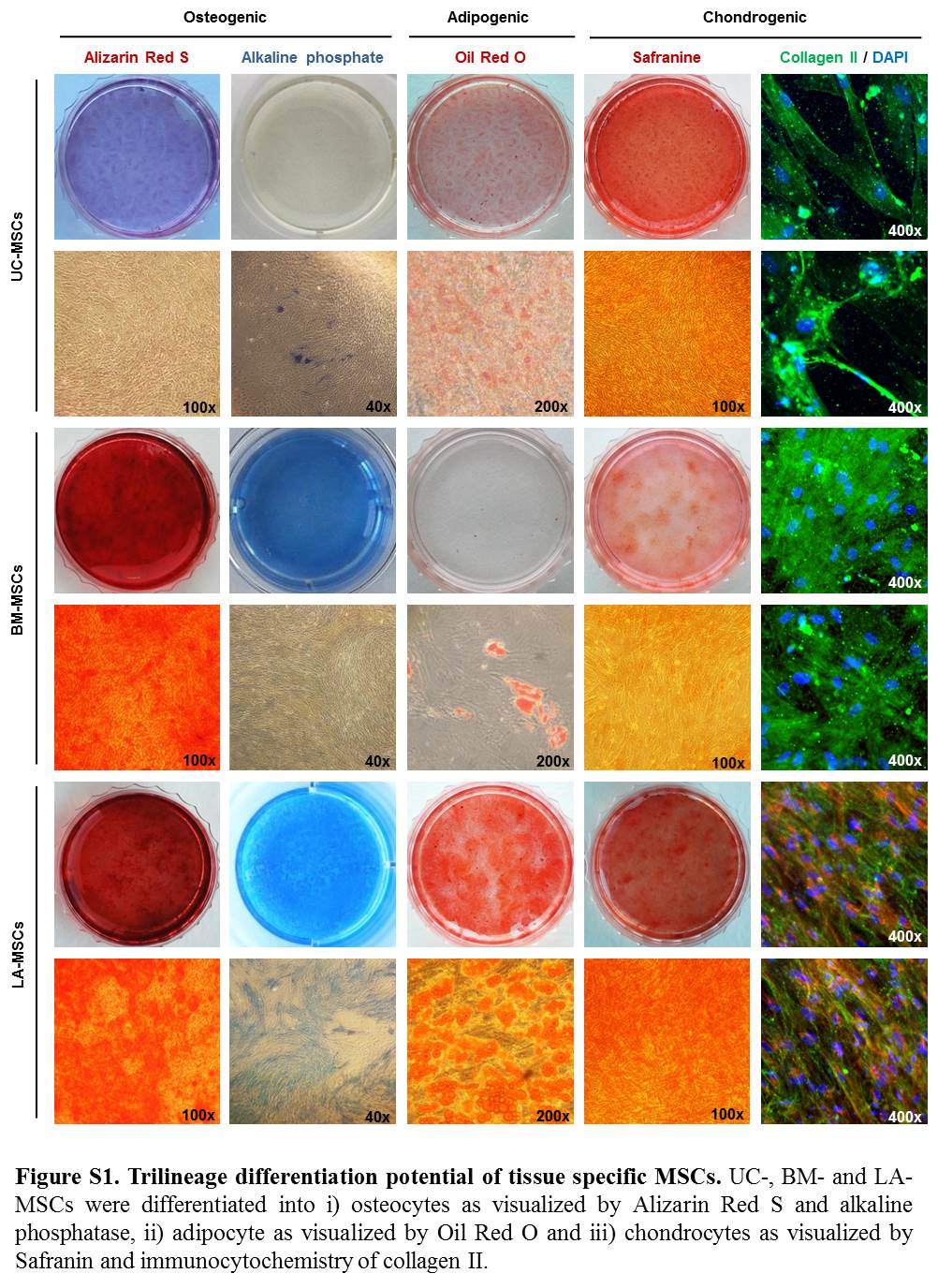

Supplement: Supplementary file 1 [file ijms-21-05366-s001.zip › Figure S1.jpg]

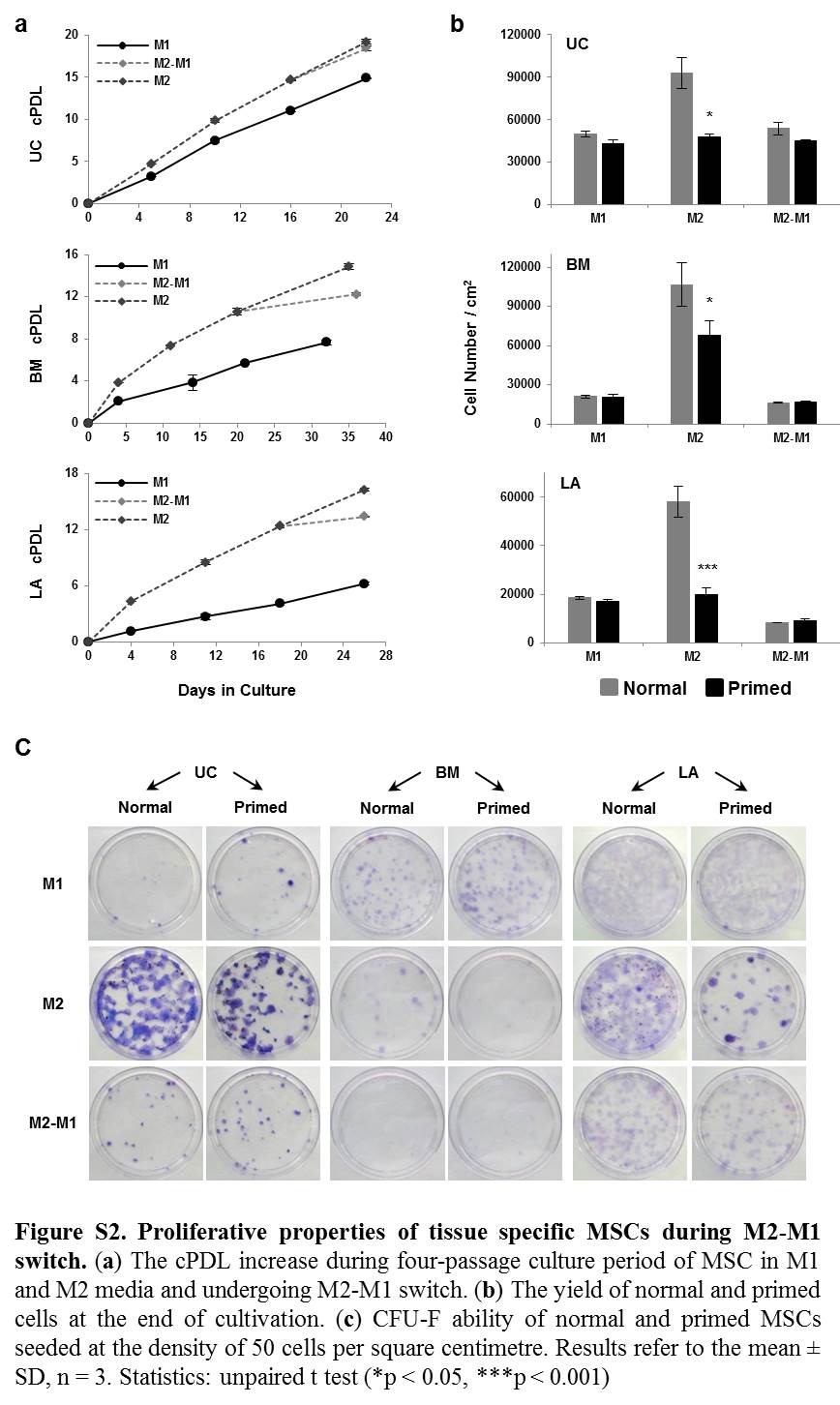

Supplement: Supplementary file 1 [file ijms-21-05366-s001.zip › Figure S2.jpg]

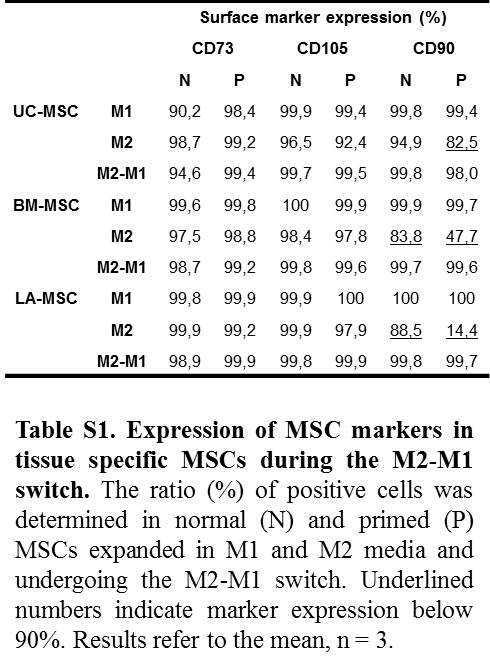

Supplement: Supplementary file 1 [file ijms-21-05366-s001.zip › Table S1.jpg]
